# Factors motivating lebanese youth to adopt COVID-19 good practices: a cross-sectional study

**DOI:** 10.3389/fpubh.2023.987187

**Published:** 2023-06-29

**Authors:** Imad Bou-Hamad, Reem Hoteit

**Affiliations:** ^1^Department of Business Information and Decision Systems, Suliman S. Olayan School of Business, American University of Beirut, Beirut, Lebanon; ^2^Clinical Research Institute, Faculty of Medicine, American University of Beirut, Beirut, Lebanon

**Keywords:** COVID-19, youth, health worries, social wellbeing, media trust, good practices

## Abstract

**Background:**

It is now widely acknowledged that young people can be asymptomatic carriers of the COVID-19 virus. While vaccines are successful, COVID-19 good practices continue to be useful in controlling the virus transmission. This study aimed to investigate the associated risk factors impacting the youths' adoption of COVID-19 good practices in Lebanon.

**Methods:**

Data were collected through an online survey. The analyzed sample included 602 young people.

**Results:**

Our results indicate that around half the youth sample in our study adhere to COVID-19 good practices. COVID-19 good practices are more likely to be adopted by individuals who are more worried about their health and those who live with their partners. Furthermore, media trust was a significant predictor of COVID-19 good practices.

**Conclusion:**

Media can play a larger role in promoting good practices through youth-targeted programs. By identifying community influencers and encouraging peer-to-peer communication, it is possible to engage youth who distrust the media and persuade them to adopt COVID-19 good practices.

## Introduction

Coronavirus disease 2019 (COVID-19) has been the world's most emerging infectious disease caused by a highly contagious and pathogenic viral infection (1–3). COVID-19 has generated a slew of problems that have never been seen before among this generation. As a global pandemic, this disease prompted countries to take measures to halt its spread, negatively impacting the global economy (4, 5). As of April 2023, the World Health Organization (WHO) estimated that more than 760 million COVID-19 confirmed cases have occurred, with over 6.8 million deaths and 13 billion vaccination doses administered worldwide (6).

With new strains threatening future outbreaks, like the latest identified highly transmissible Omicron variants as we write, on 8 April 2023 (7, 8), it has been indicated that the journey to swift recovery would be long and that there will likely be numerous waves of infection (9–11). As a result, proper practices should be pursued until a high level of universal immunity is acquired.

The main tool in controlling the transmission of coronavirus at the community and individual levels is the effective implementation of preventive measures or good practices (1, 12). According to the literature, wearing masks and gloves, hygiene practices, social distancing, travel and movement restrictions, isolation, and quarantine are all essential measures for controlling and decreasing the virus's transmission (13, 14).

There has been a lot of debate about whether youth are susceptible to COVID-19. Youth, according to the WHO, are those between the ages of 15 and 24 (15). With asymptomatic and less serious illnesses, youth were found to have a reduced risk of COVID-19 morbidity and mortality (16, 17). Asymptomatic youngsters, on the other hand, may act as “silent spreaders” without getting ill, thereby indirectly contributing to the pandemic spread (18). Since Lebanese society has a higher percentage of youth than the global norm (19), the goal of this research is to investigate COVID-19 practices among young Lebanese people. We specifically seek to answer the following research question: What factors influence COVID-19 good practices among Lebanese youth?

### Background on lebanon

Lebanon is a middle-income country in the Middle East. On 21 February 2020, the first case was confirmed in Lebanon. In an attempt to flatten the curve, the Government of Lebanon implemented several lockdowns between 2020 and 2021, allowing authorities legislative authorization to implement exceptional measures against COVID-19, including travel restrictions and closures of public and private establishments (20). By April 2023, the Lebanese Ministry of Public Health (MOPH) had confirmed more than one million infections and approximately 10,800 coronavirus-related deaths in the country since the start of the COVID-19 pandemic (21).

In Lebanon, a few modeling studies were conducted to estimate the spread of SARS-CoV-2 and the impact of COVID-19 vaccination (22–24). A modeling study conducted in early April 2021 predicted a significant increase in the number of cases and deaths if schools and universities reopened, especially since the vaccination rates were below 4% (24). Additionally, El Deeb (22) found that the poverty rate is not statistically important to the spatial diffusion of the disease, whereas geographic boundaries, distance between district centers, number and density of population, and poverty density lead to disease clustering. Another study, which used a novel compartmental model to account for the effects of vaccine efficacy, deployment rates, and deployment timing, predicted that, at the same daily deployment rate, earlier introduction of vaccination schemes with lower efficacy would also reduce mortality compared to a delayed introduction of high efficacy vaccines, which can still achieve lower numbers of infections and better herd immunization (23).

Despite all attempts to promote vaccine uptake, including the national COVID-19 vaccination plan, which sought to vaccinate 70% of the population by the end of 2022, the country is still well short of fulfilling this objective by 2023 (25, 26). Vaccine coverage for the first dose was 50.4% as of 8 April 2023, with coverage for the second dose at 44.4%. Only 27.6% of those who received the second dose got the third (27).

As of 8 April 2023, there are still cases of COVID-19 infections, with an average of 100 new cases being reported per day. This time, however, it is unprecedented as the country is in the midst of a devastating crisis with physicians and nurses fleeing the country owning to the worsening financial and political instability that has plagued the country for decades (28, 29). It is of note that the COVID-19 pandemic worsened Lebanon's severe and ongoing economic and financial crisis, which started in October 2019 (25). In March 2023, the Lebanese lira was devalued by more than 95%, making this economic and financial crisis one of the worst to hit the world since the middle of the 19th century, according to the World Bank (30, 31). Due to the economic downturn, Lebanese households are having trouble getting access to fundamental services including food, healthcare, education, and others (30).

Furthermore, the overwhelming number of Syrian refugees, estimated at 1.5 million, has placed significant pressure on the country's already frail healthcare system and providers due to a shortage of international assistance (25, 32). Excluding the Palestinians and waves of Syrians who entered after 2009, youth account for 27.4% of Lebanon's overall resident population (19).

The healthcare system has continued to face issues as a result of these crises, ranging from a shortage of resources to an exponential increase in the number of patients, placing the country in peril and resulting in an overwhelmed medical system (28, 29). As a result, now more than ever, a focus on good practices is required to limit the spread of the virus, especially among youth. In comparison to the global average, Lebanon has a higher percentage of youth (19, 33) accounting for 28% of the overall population (34). Thus, this study aimed to determine whether socio-demographic characteristics, health and social wellbeing concerns, and media trust are significant predictors of COVID-19 good practices among youth.

### COVID-19 practices and demographics

COVID-19 practices were shown to be associated with a variety of factors. Male youth were shown to be less likely to practice preventive measures in a recent Ethiopian study (1). According to the same study, it was found that preventive interventions were more likely to be practiced by older youth who had a better education level and a higher income (1). In addition, a study conducted in Saudi Arabia revealed that higher practice scores were linked with women and high-income individuals, whereas lower practice scores were associated with youth (13). Female sex, older age, higher education, greater family wealth, urban residency, and having more positive views were all linked to more frequent preventative practice factors, according to a study conducted in Bangladesh (35). As such, we test the following hypotheses:

*H1: Female youth adhere to COVID-19 good practices than male youth*.
*H2: Youth with a higher degree of education are more likely to adhere to COVID-19 good practices*.
*H3:* Y*outh from higher income families are more likely to adhere to COVID-19 good practices*.
*H4: Youth who live in larger cities/towns are more likely to adhere to COVID-19 good practices*.

### Worries and safety behavior

According to various studies, perceived risk and fear of COVID-19 have been linked to good practices and a favorable attitude toward COVID-19 controllability (36, 37). Ali et al. (36) indicated that individuals from the Middle East had the highest fear score in a cross-cultural study. Additionally, a study in Croatia found a significant increase in participants' worry and safety behaviors, with persons with chronic health conditions expressing even more concern and safety behavior than healthy participants (38). Moreover, overestimation of anxiety sensitivity to COVID-19 concerns was found to be a major predictor of COVID-19-related fear and safety behaviors in a study conducted in Pakistan (39). As a result, we hypothesize the following:

*H5: Youth who are more concerned about their health are more likely to adhere to COVID-19 good practices*.
*H6: Social wellbeing concerns are associated with COVID-19 good practices*.

### Media and health intervention

The media, on the other hand, is the most important tool for health intervention (40). According to the literature, the use of mass media to change human health behaviors has been demonstrated to be effective (41). The internet has evolved into a vital global source of health-related information, and the capacity to distribute information fast during a pandemic has proven to have various benefits, including allowing health systems to prepare for the outbreak and allowing individuals to comprehend the severity of the risk (42). Based on the literature, people who have a higher level of trust in the media are more inclined to embrace health messages and practice healthy habits (43). Consequently, we expect the following hypothesis:

*H7: Youth who believe the media is a trustworthy source of information are more likely to adhere to COVID-19 good practices*.

## Methodology

### Study design and data collection

Our youth data were retrieved from a larger publicly available dataset utilized by Bou-Hamad et al. (20). The original dataset is a cross-sectional dataset gathered via an online survey utilizing the iCode smart test (20, 44). The iCode test reveals people's reluctance to share their thoughts and is a simplified version of the implicit association testing (IAT) technique, which is widely used in psychology (45). More specifically, the original data were intended to serve an international COVID-19-related project on understanding people's feelings, worries, behaviors, and opinions on the pandemic in 20 countries, including Lebanon. The project was launched by a global research and technology institute, and the questionnaire was evaluated by eight experts from the US, Poland, Singapore, Hong Kong, Portugal, and Switzerland in the fields of psychology, sociology, marketing, and economics.

A sample of 988 Lebanese was collected between 3 and 31 May 2020. The survey was made available in three languages (Arabic, English, and French), reflecting the country's diverse communication approaches. The questionnaire was delivered online via social media sites such as Facebook, LinkedIn, and WhatsApp to maintain social distance and decrease virus dissemination. An approval was received from the Lebanese American University Institutional Review Board. Data collection was performed in accordance with the Declaration of Helsinki. All appropriate measures were taken to ensure participants' anonymity and information confidentiality. Prior to filling out the survey, all participants provided informed consent online.

Respondents were shown a series of statements and asked to indicate whether they agree with the sentence on the screen. Participants were given three options: “YES,” “HARD to TELL,” and “NO.” It is worth mentioning that responses including “Hard to tell” were classified as missing values and hence excluded from the analysis. The explicit and implicit replies were recorded concurrently, with implicit responses represented by reaction time (RT). The RT measurement is based on Fazio's theory of attitude accessibility (46). This method can reveal how confident respondents are in their “Yes” (“High Yes”) or “No” (“High No”). The complete methodology on this subject can be found in Bou-Hamad et al. (20).

Since the goal of this study is to investigate youth behavior in relation to COVID-19 practices, we selected respondents aged 18 to 25 years from the data. As a result, 602 youths were included in the sample.

### Measures

Aside from demographics, there were 15 statements (Table 2) related to health worries (4 statements), social wellbeing concerns (4 statements), and good COVID-19 practice (7 statements). Responses to the above statements were converted to a 4-Likert scale as follows: 1 = “High NO,” 2 = “Low NO,” 3= “Low YES,” and 4 = “High YES.” In terms of media trust, respondents answered “yes” or “No” to the statement “Media provide reliable information about the pandemic.” The health worries, social wellbeing concerns, and practice constructs employed in this study are the same as those used by Bou-Hamad et al. (20).

Statements on COVID-19 health worries were obtained from the Coronavirus Health Impact Survey (CRISIS) (47). The CRISIS survey was utilized by Liu et al. (48) to explore people's health worries about themselves, their friends, and family members becoming infected by the virus. The three statements measuring life satisfaction, on the other hand, match an altered shortened version of the Multidimensional Students' Life Satisfaction Scale (MSLSS), which had already been introduced and used in earlier studies (49, 50). Because social isolation is inversely correlated with wellbeing (51, 52), we constructed a social wellbeing concerns variable with four variables related to pandemic isolation. The COVID-19 practice statements correspond to the items used by Ferdous et al. (35) and are consistent with the COVID-19 infection prevention and control measures recommended by the World Health Organization (53).

We validated the internal consistency of the construct items using Cronbach's alpha measure. Cronbach's alpha values for practice, health concerns, and social wellbeing concerns were 0.58, 0.68, and 0.70, respectively. An alpha between 0.6 and 0.7 indicates a satisfactory level of reliability (54). We computed scores for each construct by averaging the individual scale item scores. In the Result section, we present summary statistics for the variables studied.

### Statistical analysis

For descriptive statistics, summary measures such as proportions and frequencies are generated. In this study, the dependent variable (practice) is considered numerical. To test our hypotheses, we used ordinary least squares (OLS) regression. The robust standard errors approach is used to correct heteroscedasticity because the constant-variance assumption (homoscedasticity) was violated. The analysis is carried out using the R programming language (version 3.4.4). The statistical significance level was set at 0.05.

## Results

### Descriptive statistics

Table 1 shows the sociodemographic characteristics of the participants. Our sample included people with different levels of education and income. Nearly two-thirds of the participants were female youth (58.1%). The majority of participants (86.4%) have a bachelor's degree or higher or are enrolled in university, with one-third residing in large cities (more than 100 000 inhabitants). More than one-third of the participants (37%) had a monthly household income below 2,500,000 L.L (1 USD = 9,000 LBP in May 2020). Furthermore, 10% of the population has more than one child. In terms of employment status, 15% were employed, 74% were students, and 9% were unemployed.

**Table 1:** Sociodemographic characteristics of the study participants (*N* = 602).

**Variable**	***n* (%)**
**Gender**	
Men	233 (38.7)
Women	350 (58.1)
Missing	19 (3.2)
**Education level**	
Elementary school	4 (0.7)
Brevet degree or 9th grade	5 (0.8)
BACC II or high school	52 (8.6)
Vocational	21 (3.5)
Bachelor's degree or higher	520 (86.4)
**Living status**	
Living alone	88 (14.6)
Living with a partner	83 (13.8)
Living with parents/relatives	431 (71.6)
**Children**	
No	544 (90.4)
≥1	58 (9.6)
**City size (people)**	
< 10,000	188 (31.2)
Between 10,000 and 14,999	110 (18.3)
Between 15,000 and 99,999	46 (7.6)
Between 20,000 and 49,999	41 (6.8)
Between 50,000 and 99,999	40 (6.6)
≥100,000	177 (29.4)
**Employment status**	
Employed	92 (15.3)
Student	448 (74.4)
Entrepreneur	8 (1.3)
Unemployed	54 (9.0)
**Monthly income (LBP** ^‡^ **)**	
≤ 600,000	85 (14.1)
Between 600,000 and 1,000,000	55 (9.1)
Between 1,000,000 and 2,500,000	83 (13.8)
Between 2,500,000 and 5,500,000	37 (6.1)
Between 5,500,000 and 8,500,000	16 (2.7)
≥8,500,000	13 (2.2)
Missing	313 (52.0)

^‡^LBP is Lebanese pound (1 USD = 9,000 LBP in May 2020).

**Figure 1:**
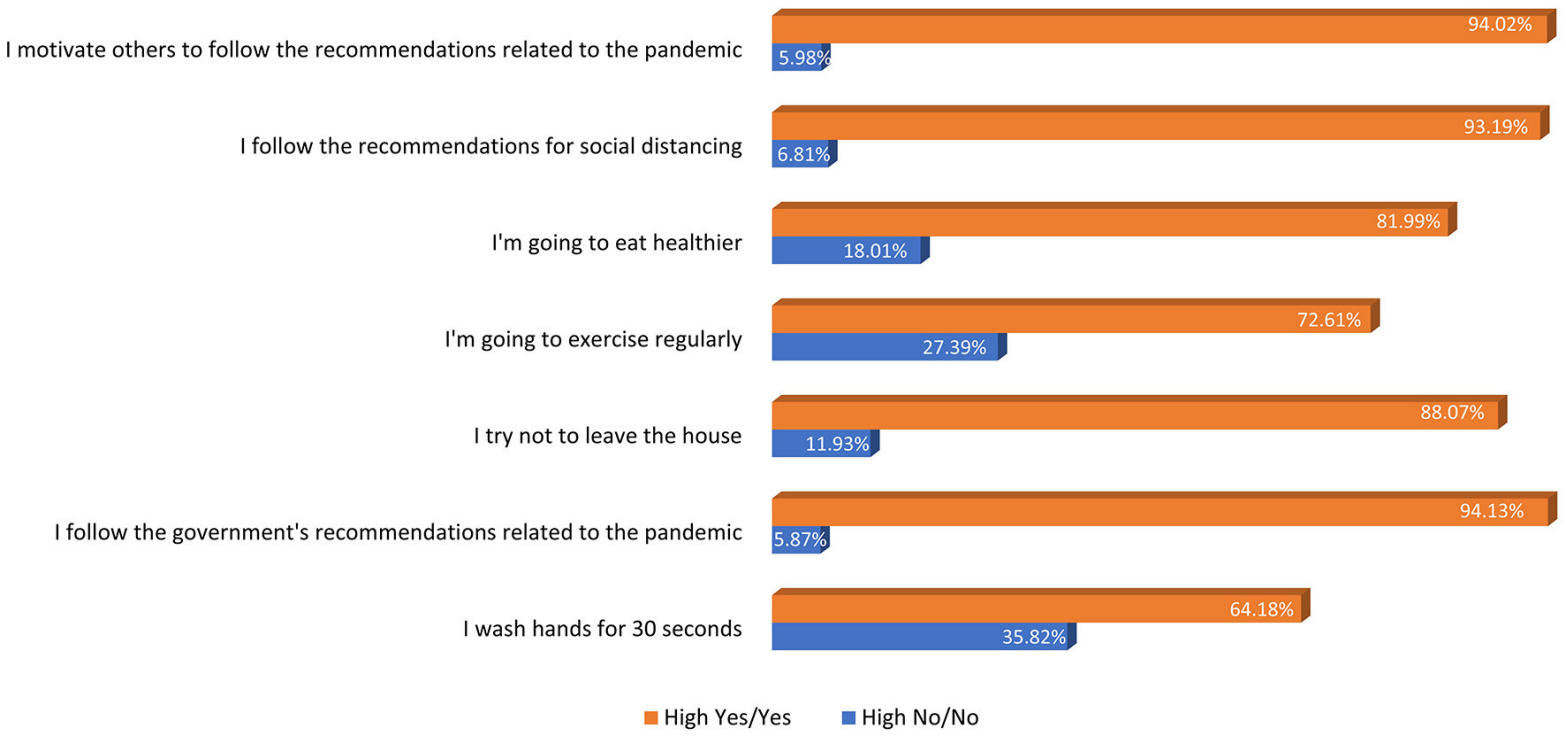
COVID-19 good practices among youth.

**Table 2** shows the percentage and frequency of responses in terms of confidence level, for each construct statement. In terms of health concerns, participants indicated that the health of their older adults family members is the most concerning issue (94% Yes and High Yes). Respondents who were concerned about not being able to meet up with friends expressed greater social wellbeing concerns (66% Yes and High Yes). Following the government's guidelines regarding the pandemic, following the recommendations for social distancing, and motivating others to follow the COVID-19 recommendations were the most common COVID-19 practices (94% Yes and High Yes) (Figure 1). Based on confident responses (High Yes), the weighted proportion of good practices among respondents was 51%. Finally, 43.7% of respondents believe that the media is a reliable source of information about the pandemic. It is important to note that the percentage of answers “Hard to tell” on this statement (media trust) was slightly higher than on other statements. More specially, 151 participants out of 602 answered “hard to tell.” Based on 451 participants, the 43.7% “Yes” response rate for media trust was calculated.

**Table 2:** Mental wellbeing outcomes and COVID-19 practices among participants.

**Variables**	**High No *n* (%)**	**No *n* (%)**	**Yes *n* (%)**	**High Yes *n* (%)**	**Hard to tell *n* (%)**
**Health worries**					
I am afraid of becoming infected with coronavirus	123 (20.4)	63 (10.5)	108 (17.9)	263 (43.7)	45 (7.5)
I am worried about my own health	90 (15.0)	92 (15.3)	125 (20.8)	265 (44.0)	30 (5.0)
I am worried about the health of my children	140 (23.3)	83 (13.8)	84 (14.0)	152 (25.2)	143 (23.8)
I am worried about the health of my older family members	6 (1.0)	12 (2.0)	148 (24.6)	420 (69.8)	16 (2.7)
**Social wellbeing concerns**					
I am worried about not being able to meet with friends	51 (8.5)	111 (18.4)	186 (30.9)	210 (34.9)	44 (7.3)
I am worried about not being able to meet with my family	81 (13.5)	155 (25.7)	185 (30.7)	146 (24.3)	35 (5.8)
I am worried that living in isolation will negatively affect my wellbeing	67 (11.1)	146 (24.3)	186 (30.9)	156 (25.9)	47 (7.8)
I am afraid that life in isolation will negatively impact my health	88 (14.6)	136 (22.6)	158 (26.2)	172 (28.6)	48 (8.0)
**Practice**					
I wash my hands for 30 s	42 (7.0)	155 (25.7)	250 (41.5)	103 (17.1)	52 (8.6)
I follow the government's recommendations related to the pandemic	13 (2.2)	21 (3.5)	215 (35.7)	330 (54.8)	23 (3.8)
I try not to leave the house	23 (3.8)	47 (7.8)	228 (37.9)	289 (48.0)	15 (2.5)
I am going to exercise regularly	75 (12.5)	57 (9.5)	121 (20.1)	229 (38.0)	120 (19.9)
I am going to eat healthier	48 (8.0)	46 (7.6)	122 (20.31)	306 (50.8)	80 (13.3)
I follow the recommendations for social distancing	25 (4.2)	14 (2.3)	94 (15.6)	440 (73.1)	29 (4.8)
I motivate others to follow the recommendations related to the pandemic	13 (2.2)	22 (3.7)	268 (44.5)	282 (46.8)	17 (2.8)

### COVID-19 practices

Table 3 represents the factors associated with COVID-19 good practices. The estimated coefficients with their statistical significance are reported. As the table shows, health worries, media trust, and living status (*p* < 0.05) are all positively associated with COVID-19 good practices. For example, individuals with higher health worries are more likely to follow COVID-19 good practices (β = 0.203, *p*-value = 0.022). Furthermore, people who live with a partner compared to those living with their parents/relatives (β = 0.315, *p*-value = 0.017) and those who believe the media provides accurate information on the pandemic (β = 0.254, *p*-value = 0.010) have better COVID-19 practices. As a result, H5 and H7 are supported. Gender, education, city size, monthly income, employment status, and social wellbeing concerns were found not to be associated with COVID-19 good practices. Consequently, the data did not provide sufficient evidence to support the hypotheses H1, H2, H3, H4, and H6. Moreover, we used the variance inflation factor (VIF) metric to determine whether there was any evidence of multicollinearity among the independent variables. The VIFs, on the other hand, were < 2. As a result, the multicollinearity issue does not exist.

**Table 3:** Regression analysis for COVID-19 good practice.

	**β**	**SE**	***t*-value**	***P*-value**
Gender^†^	0.065	0.086	0.762	0.448
City size	−0.002	0.022	−0.093	0.926
Education	−0.092	0.067	−1.373	0.174
Monthly income	−0.059	0.038	−1.564	0.121
**Employment status** ^§^				
Employed	0.062	0.179	0.348	0.728
Student	−0.091	0.170	−0.536	0.594
Entrepreneur	0.288	0.260	1.111	0.270
**Living status** ^¥^				
Living alone	0.071	0.101	0.707	0.482
Living with a partner	0.315	0.130	2.433	0.017^*^
Social wellbeing concerns	−0.044	0.078	−0.567	0.572
Health worries	0.203	0.087	2.331	0.022^*^
Media trust	0.254	0.096	2.639	0.010^*^

β Beta(s) are regression coefficients; SE standard errors are shown in parentheses, *p* is the *p*-value. ^†^Woman was used as a reference group for gender. ^§^Unemployed was used as a reference group for employment status. ^¥^Other (living with parents/relatives) was used as a reference for living status. ^*^p-value is significant (< 0.05).

## Discussion

The COVID-19 pandemic has had a significant social and mental health impact on young people (55), and as COVID-19 variants proliferate, more young people are being admitted to hospitals (56). As a result, today more than ever, a focus on good practices is necessary to minimize the virus's transmission. Our results indicate that around half the youth sample in our study adheres to COVID-19 good practices. Considering the likelihoods of youths' adherence to preventive measures are weaker, this study was conducted to investigate the associated risk factors impacting the youths' adoption of COVID-19 good practices.

Studies in the literature indicated that gender, education, income, and city size were found to be associated with good practices among youth (1, 13, 35, 57). However, our findings did not show any significant associations. Thus, none of our hypotheses about these sociodemographic variables (H1, H2, H3, and H4) were supported.

The concept of health worries matters and extends beyond one's health. A study conducted by Barragan and Meltzoff revealed that certain people have a strong desire to look out for the health of strangers, which is strongly correlated with their concern for all of humanity rather than just their own community or nation (58). Our data also supported H5 and indicated that individuals with higher health worries are more likely to follow COVID-19 good practices. There is insufficient research on the link between health worries and COVID-19 good practices among youth in the literature. Despite this, published research has related COVID-19′s perceived risk and fear to preventative or good practices (36), with individuals with chronic health conditions expressing even more concern and safety behavior than healthy participants (38). Our findings, therefore, support our hypothesis that youth who are more worried about their health are more likely to follow COVID-19 good practices.

People of all ages were affected by the COVID-19 quarantine. Social interactions and academic practices were impacted by remote study (59). We hypothesized that COVID-19 good practices are linked to social wellbeing concerns. Nonetheless, our findings revealed no statistically significant association. As a result, we failed to support this hypothesis (H6).

While recent research failed to demonstrate that being a young person living with a partner has an influence on adherence to COVID-19 good practices (60), the current study was able to support this association. More specially, we found that young people who live with a partner have greater COVID-19 good practices than others.

The use of mass media to influence human health behaviors has been shown to be effective in the literature (41). Participants in our study who believe that media provides accurate information on the pandemic have better COVID-19 good practices. According to research, people who have a higher level of trust in the media are more likely to embrace health messages and implement healthy practices (43). Another study by Adam et al. (61), revealed that media trust is crucial because it is associated with people's willingness to abide by COVID-19 regulations. Therefore, our hypothesis (H7) indicating that youth who believe the media is a trustworthy source of information are more likely to follow COVID-19 good practices has been validated. For those who do not trust the media, it is recommended that identifying popular influencers in the community who have a large following among the youth and partnering with them to promote COVID-19 precautions can be a useful way to reach this group. Another option would be to promote peer-to-peer communication, as young people frequently place more trust in their friends and peers than in more established sources of information. It is, therefore, possible to foster a sense of community and raise the likelihood that they will act by encouraging them to talk with one another about COVID-19 practices and to share information and resources.

### Limitations

Some limitations apply to this study. Respondents were recruited through social media platforms for the survey, which was conducted online. Therefore, the sample was collected conveniently. However, convenience sampling has been used frequently in COVID-19 studies. While this sampling strategy cannot always guarantee that results are generalizable, it can nevertheless be a useful tool for determining the likelihood of potential associations between variables (20, 62). Furthermore, young adults account for 98% of active internet users in the Mena region (63). As a result, we can safely assume that our sample is representative of the population of internet-savvy Lebanese young adults and that our findings apply to this population. Online surveys are considered useful data collection tools during a pandemic to reduce virus transmission. Those without access to the internet, on the other hand, were unable to participate in the survey.

## Conclusion

The current study contributed to a better understanding of the risk factors associated with youths' good practices for COVID-19. Aside from health worries and living status, media trust was a key predictor of COVID-19 good practices. As a result, media should engage in more intervention attempts to promote good practices. More specifically, media can create appealing coordinated awareness campaigns and disseminate them through youth-targeted programming. Future research should incorporate more variables on youth lifestyle habits in order to identify factors that can contribute to increased adherence to good practices.

## Data availability statement

Publicly available datasets were analyzed in this study. The datasets are available upon reasonable request from the corresponding author or can be found here: http://hdl.handle.net/10938/22923.

## Ethics statement

Our study received approval from the Lebanese American University's Institutional Review Board. The Declaration of Helsinki was followed when gathering data. To maintain participant anonymity and data confidentiality, all necessary precautions were followed. All respondents gave informed consent online before beginning the survey. The patients/participants provided their written informed consent to participate in this study.

## Author contributions

IB-H created the conceptual framework. RH performed data curation and conducted the literature review. IB-H and RH analyzed and interpreted the data, contributed to the discussion, and major contributors to writing the manuscript. All authors read, revised, and approved the final manuscript.
